# Serial FLT PET imaging to discriminate between true progression and pseudoprogression in patients with newly diagnosed glioblastoma: a long-term follow-up study

**DOI:** 10.1007/s00259-018-4090-4

**Published:** 2018-07-21

**Authors:** Cyrillo G. Brahm, Martha W. den Hollander, Roelien H. Enting, Jan Cees de Groot, A. Millad Solouki, Wilfred F. A. den Dunnen, Mart A. A. M. Heesters, Michiel Wagemakers, Henk M. W. Verheul, Elisabeth G. E. de Vries, Jan Pruim, Annemiek M. E. Walenkamp

**Affiliations:** 1 Department of Medical Oncology, University of Groningen, University Medical Centre Groningen, P.O. Box 30.001, 9700 RB Groningen, The Netherlands; 2 Department of Medical Oncology, VU University Medical Centre, Cancer Centre Amsterdam, Amsterdam, The Netherlands; 3 Department of Neurology, University of Groningen, University Medical Centre Groningen, Groningen, The Netherlands; 4 Department of Radiology, University of Groningen, University Medical Centre Groningen, Groningen, The Netherlands; 5 Department of Pathology, University of Groningen, University Medical Centre Groningen, Groningen, The Netherlands; 6 Department of Radiotherapy, University of Groningen, University Medical Centre Groningen, Groningen, The Netherlands; 7 Department of Neurosurgery, University of Groningen, University Medical Centre Groningen, Groningen, The Netherlands; 8 Department of Nuclear Medicine, University of Groningen, University Medical Centre Groningen, Groningen, The Netherlands; 9 Department of Molecular Imaging, University of Groningen, University Medical Centre Groningen, Groningen, The Netherlands

**Keywords:** Glioblastoma multiforme, FLT PET, Pseudoprogression, Chemoradiotherapy, Ki67

## Abstract

**Purpose:**

Response evaluation in patients with glioblastoma after chemoradiotherapy is challenging due to progressive, contrast-enhancing lesions on MRI that do not reflect true tumour progression. In this study, we prospectively evaluated the ability of the PET tracer ^18^F-fluorothymidine (FLT), a tracer reflecting proliferative activity, to discriminate between true progression and pseudoprogression in newly diagnosed glioblastoma patients treated with chemoradiotherapy.

**Methods:**

FLT PET and MRI scans were performed before and 4 weeks after chemoradiotherapy. MRI scans were also performed after three cycles of adjuvant temozolomide. Pseudoprogression was defined as progressive disease on MRI after chemoradiotherapy with stabilisation or reduction of contrast-enhanced lesions after three cycles of temozolomide, and was compared with the disease course during long-term follow-up. Changes in maximum standardized uptake value (SUV_max_) and tumour-to-normal uptake ratios were calculated for FLT and are presented as the mean SUV_max_ for multiple lesions.

**Results:**

Between 2009 and 2012, 30 patients were included. Of 24 evaluable patients, 7 showed pseudoprogression and 7 had true progression as defined by MRI response. FLT PET parameters did not significantly differ between patients with true progression and pseudoprogression defined by MRI. The correlation between change in SUV_max_ and survival (*p* = 0.059) almost reached the standard level of statistical significance. Lower baseline FLT PET uptake was significantly correlated with improved survival (*p* = 0.022).

**Conclusion:**

Baseline FLT uptake appears to be predictive of overall survival. Furthermore, changes in SUV_max_ over time showed a tendency to be associated with improved survival. However, further studies are necessary to investigate the ability of FLT PET imaging to discriminate between true progression and pseudoprogression in patients with glioblastoma.

## Introduction

Glioblastoma (GBM) is the most common and most aggressive primary brain tumour. It accounts for more than 50% of all gliomas and has an incidence rate of 3.19 per 100.000 in the United States [1]. Current first-line treatment, consisting of maximal surgical resection followed by postoperative radiation with concomitant and adjuvant temozolomide (TMZ) therapy, has improved 2-year survival from 11% to 27% and 5-year survival from 2% to 10% [2]. However, response evaluation of this treatment in these patients is problematic because of the difficulty in distinguishing recurrent tumour (i.e. true progression) from pseudoprogression. Pseudoprogression is defined as progressive gadolinium-enhanced lesions on MRI immediately after the end of concurrent chemoradiotherapy, following stabilisation or spontaneous improvement in the contrast-enhanced lesions without further treatment other than adjuvant TMZ [3, 4]. This is observed in 28–66% of all GBM patients undergoing chemoradiotherapy, and primarily occurs within the first 3 months after completion of chemoradiotherapy [5]. The difficulty in distinguishing true progression from pseudoprogression impedes clinical decision making in these patients. In patients with pseudoprogression, standard treatment with adjuvant TMZ should be continued, whereas in patients with true tumour progression, other treatment modalities – although scarce – or palliative supportive care are more appropriate.

The use of several amino acid tracers, including ^11^C-methionine (MET), ^18^F-fluoroethyl-l-tyrosine (FET) and l-3,4-dihydroxy-6-^18^F-fluorophenylalanine (F-DOPA), for the metabolic imaging of brain tumours has been extensively explored [6–9]. Imaging studies with these amino acid tracers have provided valuable information on the identification of nonenhancing, metabolically active tumour areas, and the prediction of treatment response in patients receiving antiangiogenic therapy [10–12].

Interestingly, ^18^F-fluorothymidine (FLT) is an ^18^F-labelled thymidine analogue that is taken up preferentially by proliferating cells. FLT tracer uptake reflects thymidine kinase 1 activity, which is involved in DNA synthesis, and can be used as a measure of cell proliferation. In several tumour types, FLT uptake measured with PET corresponds to the Ki67 proliferation index, and its change is correlated with response to therapy [13, 14].

In glioma patients, FLT uptake has been used for tumour grading and is correlated with Ki67 proliferation index [15, 16]. Moreover, FLT PET has been found to perform better in predicting survival and recurrence in glioma patients than FDG PET and MRI [17, 18]. However, to date, no prospective study has been conducted to determine the ability of FLT PET to discriminate between pseudoprogression and true progression. Therefore, the aim of this prospective study in patients with newly diagnosed GBM was to determine whether FLT PET scans, performed before and after chemoradiotherapy, can discriminate between true progression and pseudoprogression as measured by MRI after three courses of adjuvant TMZ. In addition, MRI responses were compared and verified in relation to the disease course during long-term follow-up.

## Materials and methods

### Patients and treatment

Patients with newly diagnosed GBM or gliosarcoma (WHO grade IV, hereafter referred to as GBM) who were eligible for standard treatment with radiotherapy and TMZ were prospectively included. After surgical resection or biopsy, patients were treated with radiotherapy consisting of 2 Gy irradiation 5 out of 7 days per week for 6 weeks, for a total dose of 60 Gy. Patients received concomitant TMZ orally at a dose of 75 mg/m^2^ daily for 6 weeks. After a treatment break of 4 weeks, patients received up to six cycles of adjuvant TMZ (150–200 mg/m^2^) for 5 days every 28 days. The use of corticosteroids during treatment was recorded. No changes in treatment were introduced based on the results of the FLT PET scan. Overall survival was calculated from the date of informed consent to the date of death or last known date alive, censored at the time of analysis (end of December 2017). Written informed consent was obtained from all individual participants included in the study. The protocol was approved by the local medical ethics committee and registered with the Dutch trial register (NTR3680).

### MRI imaging

Patients underwent standard radiological follow-up with MRI (1.5 T using T1, T2 and contrast-enhanced 3D T1 gradient echo sequences) within 72 h of surgery (baseline), 10 weeks after the start of treatment (4 weeks after completing chemoradiotherapy), 22 weeks after the start of treatment (after the third cycle of adjuvant TMZ or earlier as clinically indicated), and every 3 months thereafter. MRI data for this study were assessed by an independent neuroradiologist and a radiologist-in-training using the Macdonald criteria for tumour response evaluation [19]. Pseudoprogression was defined as progressive disease on MRI at 10 weeks, with stabilisation or reduction in enhancing lesions on MRI at 22 weeks. True progression was defined as progressive disease on MRI at both 10 weeks and 22 weeks. The MRI responses were confirmed in relation to the disease course during long-term survival follow-up of these patients.

#### FLT PET imaging

FLT was synthesized as described by Been et al. [20]. FLT PET scans were performed after surgery, but before the start of radiotherapy (baseline) and 10 weeks after the start of treatment (4 weeks after completing chemoradiotherapy). Patients were instructed to fast for a minimum of 4 h before intravenous injection of tracer. For FLT, 200 MBq was administered 30 min before the baseline PET scan (mean ± SD 201.22 ± 14.16 MBq) and follow-up scan (mean ± SD 196.60 ± 26.70 MBq). A 60-min dynamic protocol was used in the first three patients to determine the optimal timing, followed by an abbreviated, static protocol of 30 min in the remaining patients. PET scans were performed on either an HR+ ECAT Exact or an mCT PET scanner (Siemens, Knoxville, TN). Baseline and follow-up PET scans were performed on the same scanner in almost all patients. Both the ECAT Exact and mCT PET scanners were standardized according to The Netherlands protocol for standardization and quantification of FDG whole-body PET studies in multicentre trials, which ultimately formed the foundation for the European Association of Nuclear Medicine (EANM) procedure guidelines for tumour PET imaging [21–23].

The maximum standardized uptake value (SUV_max_) was assessed according to EANM procedure guidelines by drawing a region of interest (ROI) around every lesion on a separate reconstruction [22]. For multiple lesions, the mean SUV_max_ was calculated. FLT PET scans were fused with the most recent MRI scan to differentiate actual tumour from postsurgery effects outside the cerebrum if needed. The SUV_mean_ for normal brain tissue was assessed by drawing a ROI in the contralateral brain tissue. Tumour and nontumour ROIs were drawn by the same clinical researcher and were confirmed by a nuclear medicine physician. Tumour-to-normal (T/N) ratios were determined by dividing the SUV_max_ of the tumour by the SUV_mean_ of the normal brain tissue. Threshold values for SUV_max_ and T/N ratio, and a FLT PET response, defined as a 25% decrease in SUV_max_ between the first and second FLT PET scan, were based on corresponding FLT studies in the literature [18, 24, 25].

### Ki67 immunohistochemical staining

Deparaffinized GBM tissue from primary surgery or biopsy was used to evaluate the proliferation fraction of tumour cells (tissue slices of thickness 4 μm). Antigen retrieval was performed using 10 mM Tris/1 mM EDTA (pH 9) in a microwave at 700 W. Endogenous peroxidase and biotin were blocked using routine techniques. The slides were incubated with the primary antibody, Ki67 (clone MIB-1; Dako, Glostrup, Denmark) at room temperature for 1 h, followed by application of the secondary antibody, peroxidase-conjugated rabbit anti-mouse serum (Dako), and the tertiary antibody, peroxidase-conjugated goat anti-rabbit serum (Dako), for 30 min each. The first antibody was diluted 1/100 in 1% bovine serum albumin (BSA)/phosphate-buffered saline (PBS). The secondary and tertiary antibodies were diluted 1/100 in 1% BSA/PBS with 1% AB serum. Colour was developed with 3,3′-diaminobenzidine (Sigma, Zwijndrecht, The Netherlands) for 10 min. The slides were scanned for hot spots of proliferative activity. In one high-power field (×400 magnification) the fraction of Ki67-positive nuclei/total number of nuclei was determined.

### Statistics

Taal et al. found that 18 of 85 patients (20%) had discordant MRI scans showing disease progression on the first follow-up scan 4 weeks after the end of radiotherapy followed by stabilisation or a reduction in the contrast-enhanced lesions on MRI at 22 weeks, indicating pseudoprogression [3]. McNemar’s test showed that five discordant MRI scans in the absence of discordant FLT PET scans would be sufficient to prove the superiority of FLT PET over MRI for discriminating between true progression and pseudoprogression. Based on these assumptions, at least 25 patients were needed for this study.

An independent samples *t* test and the Mann–Whitney *U* test were used to compare FLT uptake and T/N ratios, respectively, between patients with and without pseudoprogression. To discriminate between true progression and pseudoprogression, receiver operating characteristic curves were used to find an optimal cut-off value for FLT uptake and changes in uptake. Fisher’s exact test was used to determine if FLT PET could accurately identify patients with pseudoprogression, based on optimal cut-off values. Kaplan-Meier curves with the log-rank test were used to analyse survival in our long-term survival follow-up. An additional multiple Cox regression analysis was performed on survival data to correct for clinical variables (i.e. tumour extent and size, steroid use and Ki67 proliferation index). Furthermore, hazard ratios (HRs) for clinical variables were calculated and are reported with 95% confidence intervals (CIs). Lastly, a Pearson correlation test was used to calculate correlations between FLT uptake and proliferation index. A two-sided *p* value of <0.05 was considered significant. Statistics were calculated using IBM SPSS Statistics 22. Graphs were generated using GraphPad Prism version 7.02 for Windows.

## Results

### Patients

**Fig. 1:**
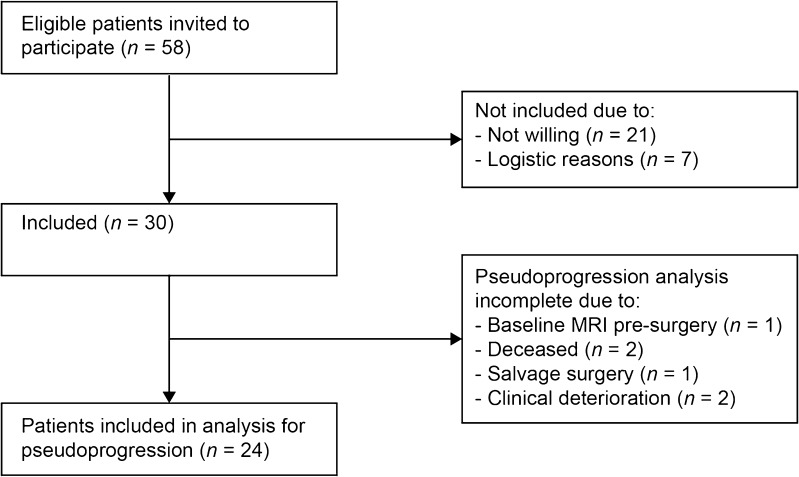
CONSORT diagram

Of 30 patients (28 with GBM and 2 with gliosarcoma, WHO grade IV) included between November 2009 and November 2012 (Table 1), five were not evaluable for pseudoprogression due to early death, salvage surgery or clinical deterioration that prevented further participation in the study, and one was excluded from the pseudoprogression analysis as only a baseline MRI scan before tumour resection was available. The CONSORT diagram is shown in Fig. 1.

**Table 1:** Patient characteristics

Characteristic	Value
Age (years), median (range)	58 (33–68)
Sex, *n*	
Male	17
Female	13
Tumour type, *n*	
Glioblastoma	26
Secondary glioblastoma	2
Gliosarcoma	2
Type of intervention, *n*	
Biopsy	3
Surgical resection	27
Completed treatment, *n*	
Radiotherapy	29
Concomitant temozolomide	23
Adjuvant temozolomide^a^	11

^a^Of 27 patients available for analysis

Baseline FLT PET scans were performed 4.9 ± 3.8 days before the start of radiotherapy, except in two patients who had their baseline FLT PET scan 2 and 4 days after the start of radiotherapy for logistic reasons. Follow-up FLT PET scans were performed 27.0 ± 8.0 days after completion of radiotherapy. Three patients had their follow-up FLT PET scan 1 day after the start of adjuvant TMZ. Finally, for logistic reasons two patients had their FLT PET scan 6 and 22 days after the start of adjuvant TMZ, respectively.

### Pseudoprogression as defined by MRI response

**Fig. 2:**
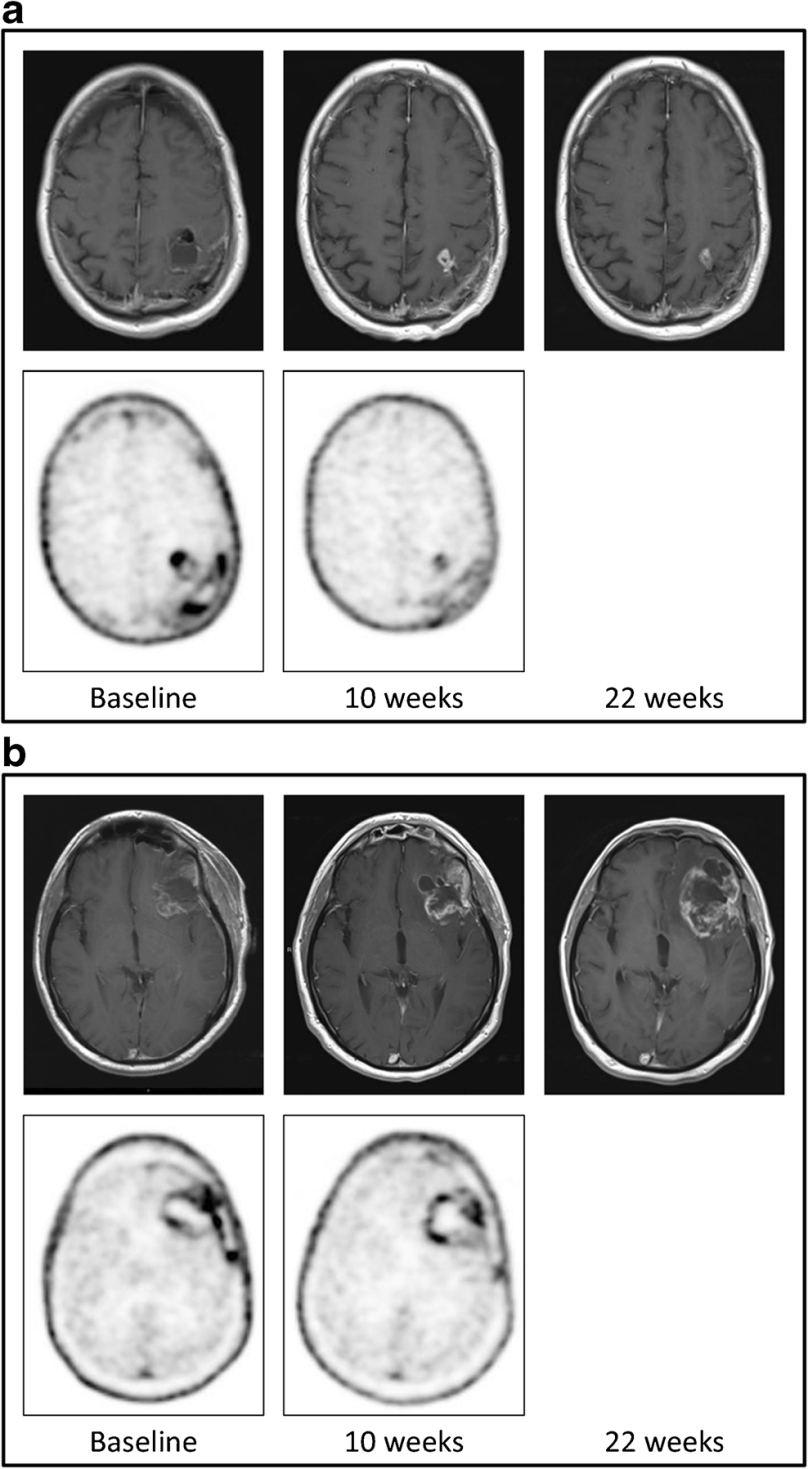
MRI and FLT PET imaging in a patient with (**a**) pseudoprogression and (**b**) true progression: *top row*: MRI images at baseline (*left*), 10 weeks (*centre*) and 22 weeks (*right*); *bottom row*: PET images at baseline (*left*) and 10 weeks (*right*). FLT PET images showed a SUV_max_ of 1.44 at baseline and 0.74 at 10 weeks in the patient with pseudoprogression, and 3.70 at baseline and 1.80 at 10 weeks in the patient with true progression

A total of 24 patients were analysed for pseudoprogression (Fig. 1). The mean SUV_max_ values at baseline and at 10 weeks in these 24 patients were 1.96 ± 1.00 and 1.28 ± 0.53, respectively. Pseudoprogression was observed in seven patients, and true progression in seven other patients (Fig. 2). Ten patients had either stable disease or a complete response on MRI after 10 weeks (Table 2). Six patients, of whom one had pseudoprogression and another had true progression, initially showed no baseline FLT uptake due to a macroscopic gross total resection of their tumour. Therefore, some of the pseudoprogression analyses had to be performed in the remaining patients.

**Table 2:** Overview of results in all included patients

Category	Patient no.	SUV_max_ baseline	T/N baseline	SUV_max_10 weeks	T/N 10 weeks	Change SUV_max_ (%)	MRI 10 weeks	MRI 22 weeks	Ki67 (%)	Overall survival (months)
Pseudoprogression	3	NU	ND	0.81	3.1	ND	PD	SD	35	31.9
	4^a^	1.73	5.6	1.58	6.3	−8.7	PD	SD	35	16.8
	14	1.23	3.0	1.14	3.5	−7.3	PD	SD	60	45.3
	15	1.90	5.3	1.61	4.0	−15.3	PD	SD	18	9.5
	18	4.17	8.7	2.67	3.3	−36.0	PD	SD	ND	9.4
	25	1.61	6.0	1.33	3.9	−17.4	PD	SD	50	41.2
	28	1.44	7.6	0.74	4.1	−48.6	PD	SD	50	50.2^c^
Progressive disease	8	2.18	14.5	0.74	4.4	−66.1	PD	PD	50	19.3
	13	NU	ND	0.96	1.7	ND	PD	PD	25	8.8
	20	1.38	3.9	1.68	2.8	21.7	PD	PD	30	11.3
	21	1.59	5.9	0.85	2.3	−46.5	PD	PD	25	59.4^c^
	22^a,b^	0.65	2.0	0.68	2.7	4.6	PD	PD	20	13.6
	27	3.70	9.5	1.80	5.3	−51.4	PD	PD	50	5.9
	29	2.93	8.1	2.23	5.9	−23.9	PD	PD	7	7.5
Other	2	NU	ND	NU	ND	ND	SD	SD	40	40.3
	5	1.24	3.1	1.46	3.2	17.7	SD	PD	40	19.7
	6^a^	1.75	4.4	0.95	2.9	−45.7	SD	SD	30	31.2
	9	2.43	6.2	1.14	2.5	−53.1	SD	SD	30	24.0
	10	NU	ND	NU	ND	ND	CR	PD	10	14.1
	11^a^	NU	ND	NU	ND	ND	CR	CR	50	28.4
	16	3.00	5.9	1.26	2.6	−58.0	SD	PD	19	9.7
	19	1.10	2.0	1.00	1.9	−9.1	SD	SD	40	62.2^c^
	23^a,b^	0.35	1.5	NU	ND	ND	CR	CR	15	19.4
	26	2.85	16.8	0.93	5.5	−67.4	SD	PD	60	6.4
Excluded from analysis	1	1.55	3.4	1.33	2.8	−14.2	NE	SD	30	29.0
	7	1.59	7.6	1.34	8.4	−15.7	PD	PD	25	10.0
	12^a^	2.84	9.8	1.64	5.1	−42.3	SD	ND	30	4.1
	17	5.02	9.0	ND	ND	ND	ND	ND	50	1.3
	24	1.64	6.6	ND	ND	ND	SD	ND	20	8.7
	30	1.42	3.3	ND	ND	ND	SD	SD	ND	10.6

*CR* complete response, *PD* progressive disease, *SD* stable disease, *ND* not done, *NE* not evaluable, *NU* no uptake

^a^Patient underwent follow-up FLT PET during adjuvant temozolomide treatment (range 1–22 days)

^b^Patient underwent baseline FLT PET during radiotherapy (range 2–4 days)

^c^Patient censored at date last known alive

Patients with pseudoprogression had mean SUV_max_ values of 2.01 ± 1.08 at baseline and 1.41 ± 0.65 at 10 weeks, compared with 2.07 ± 1.11 at baseline and 1.28 ± 0.62 at 10 weeks in patients with true progression. There was no significant difference between patients with pseudoprogression and those with true progression in SUV_max_ at baseline (*p* = 0.928), SUV_max_ at 10 weeks (*p* = 0.699), change in SUV_max_ (*p* = 0.567) and T/N ratio (*p* = 0.699) on FLT PET scans. Furthermore, FLT parameters in patients with pseudoprogression and those with true progression did not significantly differ from the FLT parameters in patients with stable disease or complete response.

Two of the patients with pseudoprogression were identified based on FLT uptake reduction, while three patients with true progression also showed a decrease in SUV_max_ of more than 25% (sensitivity 29%, specificity 43%). Furthermore, cut-off values identified as optimal by others for identifying recurrent tumour with a SUV of ≥1.34 and a T/N ratio of ≥4.94 were applied to FLT PET scans at 10 weeks [24, 25]. However, this approach did not provide an accurate prediction in all patients. ROC curves showed no other reasonable cut-off value for any parameter.

### Long-term follow-up

In all 30 patients, a baseline FLT PET scan was available. However, five patients showed no FLT uptake on baseline FLT PET. Therefore, survival analyses with SUV_max_ at baseline were based on 25 patients. At the end of December 2017, 27 patients had died and three were censored at the date last known to be alive. The median overall survival in all patients was 14.1 months (95% CI 3.4–24.8 months).

SUV_max_ at baseline and 10 weeks were both significantly correlated with survival (HR = 3.03, 95% CI 1.72–5.33. *p* < 0.001, and HR = 5.16, 95% CI 1.83–14.55, *p* = 0.002, respectively). The correlation between change in SUV_max_ (∆SUV_max_) and survival almost reached the standard level of statistical significance (HR = 0.44, 95% CI 0.19–1.03, *p* = 0.059). When compared to the response defined by MRI after three cycles of adjuvant TMZ, MRI response was more significantly associated with survival (*p* = 0.028) than SUV_max_ at baseline (*p* = 0.048) and at follow-up (*p* = 0.044).

Furthermore, use of steroids, tumour size and extent of disease were significantly associated with survival (*p* = 0.007, *p* = 0.001 and *p* = 0.047, respectively). After correction for these clinical variables, SUV_max_ at baseline remained significantly correlated with survival (HR = 6.82, 95% CI 1.31–35.42, *p* = 0.022; Table 3). Furthermore, the results of the subgroup analysis, excluding six patients who were scanned during radiotherapy or TMZ treatment, were comparable to those of the main analysis.

**Table 3:** Univariate and multivariate survival analyses

		No. of events/no. of patients	Hazard ratio (95% CI)	*p* value
Univariate analysis				
SUV_max_ at baseline		–	3.03 (1.72–5.33)	<0.001
SUV_max_ at 10 weeks		–	5.16 (1.83–14.55)	0.002
∆SUV_max_		–	0.44 (0.19–1.03)	0.059
Use of steroids	No	8/10	1	0.007
	Yes	19/20	3.21 (1.37–7.53)	
Tumour size (mm^2^)		–	1.00 (1.000–1.001)	0.001
Tumour extent	Single lobe	19/22	1	0.047
	Multiple lobes	8/8	2.37 (1.01–5.55)	
Ki67 (%)		–	0.97 (0.94–1.01)	0.095
Multivariate analysis				
SUV_max_ at baseline		–	6.82 (1.31–35.42)	0.022
SUV_max_ at 10 weeks		–	5.01 (0.62–40.56)	0.131
∆SUV_max_		–	0.42 (0.12–1.45)	0.170
Use of steroids	No	8/10	1	0.554
	Yes	19/20	1.50 (0.39–5.70)	
Tumour size (mm^2^)		–	1.00 (1.000–1.004)	0.011
Tumour extent	Single lobe	19/22	1	0.022
	Multiple lobes	8/8	18.38 (1.54–219.96)	
Ki67 (%)		–	1.01 (0.97–1.06)	0.595

### Proliferation index

In the 28 patients with specimens available for Ki67 staining, the mean SUV_max_ at baseline and at 10 weeks, and ∆SUV_max_ did not correlate with the Ki67 index of the tumour tissue before treatment (*r* = 0.233, *p* = 0.285; *r =* −0.321, *p* = 0.145; and *r* = −0.191, *p* = 0.420, respectively).

## Discussion

In this small, prospective trial, we defined pseudoprogression and true progression based on both MRI scans, and compared MRI responses with the disease course during long-term follow-up. Changes in SUV_max_ (∆SUV_max_) between the FLT PET scan at baseline and 10 weeks did not discriminate between true progression and pseudoprogression as defined by MRI. Interestingly, during long-term follow-up, ∆SUV_max_ between baseline and 10 weeks showed a tendency to be associated with improved survival. Furthermore, in the 24 patients included in our analysis, a lower baseline FLT uptake did not correlate with Ki67 index, but was predictive of a longer survival.

Despite the urgent need to distinguish between true progression and pseudoprogression in GBM patients, this is one of the few prospective studies that has assessed the ability of FLT PET imaging to distinguish pseudoprogression from true progression with long-term follow-up [26]. To date, mainly retrospective studies have been performed in patients with radiological suspicion of recurrent brain tumour at different time points, and these have shown variable results. In one study, FLT PET had a low specificity for distinguishing recurrent tumour from benign lesions in 20 patients [25]. Three other studies were able to discriminate between true progression and radionecrosis in 15, 19 and 21 glioma patients, respectively, using FLT kinetic values and the T/N ratio [24, 27, 28].

MRI is still considered the optimal modality for the assessment of treatment response and effects [3]. Consequently, changes on MRI at 10 and 22 weeks were used in our study to define pseudoprogression and true progression. Unfortunately, at the time of this study, the RANO criteria for glioma response evaluation on MRI were still under development, and therefore, the MacDonald criteria were used instead. As well as the imaging characteristics on conventional contrast-enhanced T1-weighted MRI images, the RANO response criteria also include characteristics on T2-weighted and fluid-attenuated inversion recovery (FLAIR) images [29]. However, due to the difficulty in identifying tumour lesions without contrast enhancement and the quantitative evaluation of the degree of T2/FLAIR changes to define tumour progression, an adequate assessment of treatment response or tumour recurrence with the help of the RANO criteria remains problematic [30, 31].

A key limitation of FLT, in contrast to MET, FET and F-DOPA amino acid tracers, is that FLT uptake is primarily restricted to contrast-enhancing tumour lesions due to its dependence on the permeability and tumour disruption of the blood–brain barrier [31, 32]. Therefore, the inability to accurately discriminate between true progression and pseudoprogression in our prospective study with FLT PET may well have been due to the fact that FLT uptake in high-grade gliomas reflects not only trapping of FLT in proliferating tumour cells, but also disruption of the blood–brain barrier [33]. As a result, areas showing true progression as well as pseudoprogression would show an increased FLT uptake.

An important limitation of this study is that only SUV_max_ was used for quantification of FLT uptake. The use of SUV_max_ does not take into account the heterogeneity in FLT uptake. Therefore, kinetic analysis might be of interest to distinguish between FLT uptake due to proliferation and FLT leakage that results from disruption of the blood–brain barrier, as shown in previous studies [33–36]. In addition, kinetic analysis would support the correct interpretation of the static FLT data. Unfortunately, kinetic analysis could not be performed in the present study, as FLT PET scans were performed 30 min after tracer injection. However, SUV_max_ is easy to obtain, is mostly used in clinical practice with FDG PET imaging, and has been proven to be robust. In glioma, SUV_max_ quantification of FLT uptake has a repeatability coefficient of 23%, which seems to be better than corresponding values for FDG PET [37, 38]. Furthermore, in other studies FLT kinetic values have been found to be well correlated with SUV parameters [39, 40]. Several studies have suggested other parameters for quantification of FLT PET, such as proliferative volume and parametric response maps [12, 41]. Due to the small numbers of patients and the different approaches used for quantification, direct comparison of the results is difficult.

Lastly, it is difficult to determine the optimal timing of serial FLT PET imaging before and during GBM treatment. Since the aim of this study was to differentiate between true progression and pseudoprogression after chemoradiotherapy, the baseline FLT PET scan was performed after surgery. Imaging before surgery would have revealed tumour uptake, but most patients undergo a gross total resection of tumour tissue. However, imaging after surgery can also lead to increased FLT uptake due to increased blood flow and proliferation as part of the wound healing process. This might also explain the lack of correlation between FLT uptake and the Ki67 index in our study, in contrast to the results of previous studies, in which the FLT PET scans were often performed before surgery [15–17].

Interestingly, FLT PET uptake at baseline and at 10 weeks was significantly correlated with survival. Furthermore, a decrease in FLT uptake over time also showed a tendency to be associated with improved survival (*p* = 0.059). After correction for clinical variables, only baseline FLT uptake remained significantly associated with survival. However, previous studies have also confirmed that (change in) FLT uptake is a strong independent predictor of survival [12, 18, 42]. This is in line with the results of imaging studies using FET and F-DOPA amino acid PET tracers [11, 12]. Therefore, FLT uptake may still provide useful prognostic information in patients with GBM.

## Conclusion

Our study suggests that further evaluation of FLT PET imaging is warranted to define its ability to discriminate between pseudoprogression and true progression in GBM patients treated with chemoradiotherapy, as this remains an urgent unmet need.
